# A robust and cost-effective approach to sequence and analyze complete genomes of small RNA viruses

**DOI:** 10.1186/s12985-017-0741-5

**Published:** 2017-04-07

**Authors:** Kiril M. Dimitrov, Poonam Sharma, Jeremy D. Volkening, Iryna V. Goraichuk, Abdul Wajid, Shafqat Fatima Rehmani, Asma Basharat, Ismaila Shittu, Tony M. Joannis, Patti J. Miller, Claudio L. Afonso

**Affiliations:** 1 Exotic and Emerging Avian Viral Diseases Research Unit, Southeast Poultry Research Laboratory, US National Poultry Research Center, Agricultural Research Service, USDA, 934 College Station Road, Athens, GA 30605 USA; 2 BASE2BIO, 1945 Arlington Drive, Oshkosh, WI 54904 USA; 3 National Scientific Center Institute of Experimental and Clinical Veterinary Medicine, 83 Pushkinskaya Street, Kharkiv, 61023 Ukraine; 4 Quality Operations Laboratory (QOL), University of Veterinary and Animal Sciences, Syed Abdul Qadir Jilani Road, Lahore, 54000 Pakistan; 5 Institute of Biochemistry and Biotechnology, University of Veterinary and Animal Sciences, Syed Abdul Qadir Jilani Road, Lahore, 54000 Pakistan; 6 Regional Laboratory for Animal Influenza and other Transboundary Animal Diseases, National Veterinary Research Institute, PMB01, Vom, 930010 Plateau State Nigeria

**Keywords:** Newcastle disease virus, Next-generation sequencing, Multiplexing, Galaxy, De novo assembly, Multiplexing, Complete genomes, Mixed infection, Avian paramyxovirus

## Abstract

**Background:**

Next-generation sequencing (NGS) allows ultra-deep sequencing of nucleic acids. The use of sequence-independent amplification of viral nucleic acids without utilization of target-specific primers provides advantages over traditional sequencing methods and allows detection of unsuspected variants and co-infecting agents. However, NGS is not widely used for small RNA viruses because of incorrectly perceived cost estimates and inefficient utilization of freely available bioinformatics tools.

**Methods:**

In this study, we have utilized NGS-based random sequencing of total RNA combined with barcode multiplexing of libraries to quickly, effectively and simultaneously characterize the genomic sequences of multiple avian paramyxoviruses. Thirty libraries were prepared from diagnostic samples amplified in allantoic fluids and their total RNAs were sequenced in a single flow cell on an Illumina MiSeq instrument. After digital normalization, data were assembled using the MIRA assembler within a customized workflow on the Galaxy platform.

**Results:**

Twenty-eight avian paramyxovirus 1 (APMV-1), one APMV-13, four avian influenza and two infectious bronchitis virus complete or nearly complete genome sequences were obtained from the single run. The 29 avian paramyxovirus genomes displayed 99.6% mean coverage based on bases with Phred quality scores of 30 or more. The lower and upper quartiles of sample median depth per position for those 29 samples were 2984 and 6894, respectively, indicating coverage across samples sufficient for deep variant analysis. Sample processing and library preparation took approximately 25–30 h, the sequencing run took 39 h, and processing through the Galaxy workflow took approximately 2–3 h. The cost of all steps, excluding labor, was estimated to be 106 USD per sample.

**Conclusions:**

This work describes an efficient multiplexing NGS approach, a detailed analysis workflow, and customized tools for the characterization of the genomes of RNA viruses. The combination of multiplexing NGS technology with the Galaxy workflow platform resulted in a fast, user-friendly, and cost-efficient protocol for the simultaneous characterization of multiple full-length viral genomes. Twenty-nine full-length or near-full-length APMV genomes with a high median depth were successfully sequenced out of 30 samples. The applied *de novo* assembly approach also allowed identification of mixed viral populations in some of the samples.

**Electronic supplementary material:**

The online version of this article (doi:10.1186/s12985-017-0741-5) contains supplementary material, which is available to authorized users.

## Background

Conventional laboratory methods like enzyme-linked immunosorbent assay, nucleic acid hybridization technique, and polymerase chain reaction (PCR) are all common and inexpensive diagnostic and research tools utilized in virology [1]. However, as these assays are highly dependent on reagents (primers, probes, antibodies) developed from previously known and characterized viruses, they might be ineffective for the identification of new viral variants, new pathogens or a mixed population of pathogens that have high genetic divergence from those described previously [1, 2]. Next-generation sequencing (NGS) technologies enable large numbers of samples to undergo parallel sequencing, and can be used for the detection and characterization of multiple agents from one sample. The use of sequence-independent amplification of viral nucleic acids eliminates the need for prior knowledge of genomic sequences and provides advantages over traditional methods such as PCR amplification or microarray hybridization dependent on target-specific primers [2, 3]. NGS technologies allow screening of clinical and environmental samples for the presence of viral pathogens, including previously unknown viruses [4]. This has led to the discovery of numerous viral pathogens [4–6], including 2009 pandemic influenza A, a novel pegivirus, Canine bocavirus 3, and a novel hepacivirus [7–10].

The majority of previously employed techniques used virus enrichment prior to cDNA synthesis and library preparation, or used specific primers for amplification. Virion enrichment steps such as centrifugation, polyethylene glycol precipitation, ultrafiltration, chloroform treatment or nuclease treatment have been used for RNA viruses [3, 11, 12]. Other techniques, such as gDNA depletion and host RNA depletion to enrich for viral RNA [13] and DNase pretreatment of the allantoic fluid to enrich for viral particles, have also been described [14]. Different methods have been employed for producing cDNA following enrichment, including sequence-independent single primer amplification (SISPA) and universal primers which have been used for sequencing RNA viruses [4, 15] including Newcastle disease virus (NDV) [11] and avian paramyxovirus (APMV) 4 and 6 [16].

The genus *Avulavirus* of the family *Paramyxoviridae,* order *Mononegavirales*, consists of 14 known avian paramyxovirus serotypes (APMV 1 – 14) [17–19]. Of these, APMV-1, synonymous with Newcastle disease virus, is the most widely characterized and studied due to the economic importance of Newcastle disease (ND) caused by virulent strains of the virus. Newcastle disease is one of the most significant poultry diseases and infects both wild and domestic avian hosts. NDV has a single-stranded, non-segmented, negative-sense RNA genome consisting of six genes in order of 3’ to 5’: nucleocapsid (NP), phosphoprotein (P), matrix (M), fusion (F), hemagglutinin-neuraminidase (HN), and polymerase (L), coding for these six structural proteins and at least one additional V protein [20–22]. Newcastle disease viruses have three genome sizes – 15186, 15192 and 15198 nucleotides, and are genetically grouped into two divergent classes that are further classified in genotypes [23–25].

Newcastle disease viruses are constantly evolving and different genetic groups undergo simultaneous evolutionary changes in different geographical locations [22, 26] making the available genetic makeup information outdated. These evolutionary changes present challenges for prompt diagnosis. Some currently validated methods are target-oriented and might fail to detect new viral genetic variants [27–29]. Lack of complete genetic information for many NDV isolates further hampers the better understanding of Newcastle disease evolution and epidemiology. Furthermore, mixed viral infection are not uncommon in animals, and in the case of poultry, they are quite frequent [30]. Efficient and accurate identification of these pathogens is essential for the development of adequate disease control strategies. These challenges require an approach that provides *de novo*, rapid and high-quality genetic characterization of full-length viral genomes.

Until recently, genome sequencing of small RNA viruses, including NDV, has been performed using overlapping genome amplification with primer pairs. This approach is laborious, depends on preexisting information, and produces very low depth. NGS advances provide tools for deep sequencing of multiple viral strains in a short time. However, the simultaneous cost- and time-effective sequencing and characterization of a large number of NDV genomes has not yet been reported. The aim of the current study was to utilize sequence-independent NGS technologies applied to viral nucleic acids for the simultaneous and rapid characterization of multiple NDV genomes. We demonstrate a straightforward, efficient protocol for multiplexed sequencing using a single flow cell on the Illumina MiSeq platform coupled with a detailed customized Galaxy workflow for *de novo* assembly that allows for quick and accurate generation of near-full-length, or full-length, genome sequences of dozens of isolates, simultaneously. Furthermore, we report the efficient detection and complete sequencing of contaminant RNA viruses.

## Methods

### Virus propagation

Twenty nine NDV and one APMV-13 isolates were submitted to the Southeast Poultry Research Laboratory of the USDA in Athens, Georgia, USA. The viruses were isolated in Pakistan (*n* = 15), Nigeria (*n* = 9) and Ukraine (*n* = 6) between 2003 and 2015. Viruses were propagated in 9-to-11-day-old specific-pathogen-free (SPF) embryonating chicken eggs [31]. The background information of the 30 isolates used in the study is summarized in Additional file 1: Table S1.

### RNA isolation

RNA from each sample was extracted from allantoic fluids. Two milliliters (equal volumes of 0.25 ml) of each sample were aliquoted into 8 microtubes, each containing 0.75 ml of TRIZOL LS (Invitrogen, USA). After 5 min of incubation, 0.2 ml of chloroform was added to each tube and shaken vigorously. After 10 min of additional incubation at room temperature, tubes were centrifuged at 12000 × g for 15 min at 4 °C. The aqueous phase from all eight tubes was removed and pooled for each sample. Two milliliters of aqueous layer of each sample were treated with 4 μl Turbo DNase 2U/μl (Ambion, USA) for 15 min at 37 °C and then placed on ice. The extraction proceeded using the QIAamp® Viral RNA Mini Kit (Qiagen, USA) according to the manufacturer’s instructions. Briefly, DNase-treated aqueous phase was passed through a spin column for RNA absorption on the QIAamp silica membrane followed by washing with 2 ml of provided buffers AW1 and AW2 using the QIAvac 24 Plus vacuum manifold (Qiagen, USA). The RNA was eluted in 50 μl buffer AVE. Eluted RNA was quantified using a Qubit® RNA HS Assay Kit in a Qubit® fluorometer (ThermoFisher Scientific, USA) and stored at -20 °C until further use.

### NDV RNA capture

A set of three biotinylated oligonucleotides designed from consensus of alignment of 330 available NDV genomes were used for NDV RNA capture (Oligo 1 – 5’- AGA GAA TCT GTG AGG TAC GA/3Bio -3’ at nucleotide position 8; Oligo 2 – 5’ -TTC TCA AGT CAT CGT GAC AG/3Bio -3’ at position 5905; Oligo 3 – 5’ - CCC TGC ATC TCT CTA CAG/3Bio -3’ at position 12226) (GenBank accession number AF431744). RNA capture reactions were performed using 50 μl RNA incubated with 167 μl 6X saline-sodium phosphate-EDTA buffer (900 mM NaCl, 60 mM NaH_2_PO_4_, 60 mM Na_2_EDTA), 2 μl RNaseOUT 40 U/μl (Invitrogen, USA) and 1.5 μl 100 μM mix of the three primers. Reactions were performed at 70 °C for 5 min, followed by 15 min at 55 °C. Two hundred fifty microliters of 1X binding and wash buffer solution (2 M NaCl) containing 12.5 μl (0.05%) Sera-Mag beads (magnetic streptavidin-coated beads, GE Healthcare Life Sciences, USA) was prepared for each sample and mixed with the capture reaction products for binding. Washing was done on a magnetic stand with 500 μl of 0.5X binding and wash buffer one time and twice with bead wash buffer (5 M NaCl, 1 M Tris-HCl [pH 7.5], 0.5 M EDTA, and 0.01% Tween® 20) to remove unbound RNA. Viral RNA was recovered by adding 19 μl of 10 mM Tris-HCl (pH 7.5) to the bead mixture and incubating at 65 °C for 5 min followed by cooling on ice until the next step. In a separate experiment, a comparison with three known NDV was performed and the libraries were prepared side-by-side with and without the capture step, while all remaining steps of the library preparation were identical.

### Reverse transcription

Reverse transcription reactions were performed using the M-MLV Reverse Transcriptase (Moloney Murine Leukemia Virus Reverse Transcriptase, Invitrogen, USA) and 10 μl RNA (concentration varied from below 250 pg/μl to 55 ng/μl), 1.0 μl Random Primers mix (3 μg/μl) (Invitrogen, USA) and following manufacturer’s instruction. The cDNA products were purified using 60 μl Agencourt® RNAClean® XP beads (Beckman Coulter, USA) as per manufacturer’s instructions. Purified cDNA were recovered in 15 μl of 10 mM Tris-Cl (pH 7.5) and quantified using a Qubit® ssDNA Assay Kit (ThermoFisher Scientific, USA) on the Qubit® fluorometer.

### Library preparation, quality and quantity assessment

DNA libraries (*n* = 30, one library for each sample), were prepared for deep sequencing using 1 ng purified cDNA (0.2 ng/μl in molecular grade water) and the Nextera XT DNA Library Preparation Kit (Illumina, USA) following the manufacturer's protocol. The resulting dsDNA products were purified with 30 μl Agencourt® AMPure® XP beads (Beckman Coulter, USA) by incubating at room temperature for 5 min followed by two washes with 200 μl 80% ethanol on a magnetic stand. The tubes with the beads were air dried for 10 to 15 min at room temperature and the library products were recovered in 52.5 μl of Resuspension Buffer. The quality and fragment length distribution for each library was assessed using the Agilent High Sensitivity DNA Kit (Agilent Technologies, USA) on the Agilent 2100 Bioanalyzer (Agilent Technologies, Germany). The Qubit® fluorometer and the Qubit®dsDNA HS Assay Kit were used for measuring the concentration of the libraries.

### Equimolar dilution and pooling of the NGS libraries

All libraries for NGS were diluted to a 4 nM concentration, based on their determined concentrations and fragment sizes. Equal volumes of 5 μl of each library were pooled and denatured with NaOH (0.2 N final concentration) for 5 min. The pooled mixture was vortexed and spun briefly and incubated at room temperature for 5 min. The pool was further diluted to 20 pM concentration with chilled HT1 hybridization buffer (Illumina, USA). Using the same buffer, the final concentration of the library pool was diluted to 10 pM. Control library (3% PhiX library, Illumina, USA) was added and the pool was snap-chilled on ice. The library pool (600 μl) was loaded in the flow cell of the 500 cycle MiSeq Reagent Kit v2 (Illumina, USA) and pair-end sequencing (2 × 250 bp) was performed on the Illumina MiSeq instrument (Illumina, USA). After automated cluster generation in MiSeq, the sequencing reads were processed and all statistical data generated by the instrument were collected and summarized.

### Genome assembly

**Fig. 1:**
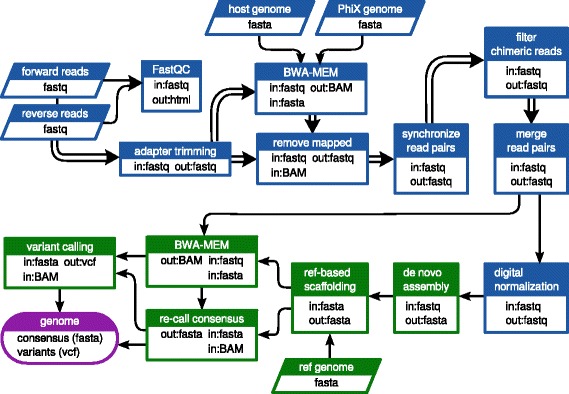
Customized Galaxy workflow used in the current study. *Double arrows* indicate steps where the read pairs were processed in parallel. *Blue shading* indicates pre-processing steps; *green shading* indicates assembly/post-processing steps; output is shaded purple. “In” indicates input filetypes; “out” indicates output filetypes

A workflow was designed to perform pre-processing and assembly of the raw sequencing data as diagrammed in Fig. 1 using the Galaxy platform interface [32]. Processing was carried out via Galaxy and PBS/Torque on a local dual node 128-core cluster. Briefly, raw read quality was assessed using FastQC [33] and residual adapter sequences were trimmed using Cutadapt v1.6 [34]. Trimmed reads were mapped against the *Gallus* and PhiX174 reference genomes using BWA-MEM v0.2.1 in order to identify host and control library read contamination [35, 36]. Host and control library reads were filtered using the Filter sequences by mapping v0.0.4 tool in Galaxy [37]. The forward and reverse files, which were no longer synchronized due to adapter trimming and filtering, were re-synchronized using in-house tool. Overlapping read pairs were joined with PEAR v0.9.6.0 [38]. Chimeric Nextera reads were removed by an in-house tool which discarded single reads with partial mappings in opposite orientations. Digital normalization via median k-mer abundance was performed using the Khmer package v1.1-1 (cutoff = 100, kmer size = 20, number of tables to use = 4, table size = 1e9) [39, 40]. *De novo* assembly was performed using the MIRA assembler v3.4.1 [41]. The following parameters and settings were specified for the assembly step: assembly method = *de* novo, assembly quality grade = accurate, use read extension = yes, minimum reads per contig = 100, minimum overlap = 16, mark repeats = yes, maximum megahub ratio = 0.2, spoiler detection = yes, with default settings for the rest of the parameters. Reference-based orientation and scaffolding of the contigs produced by the assembler were performed using V-FAT v1.0.0 (Broad Institute, Cambridge, MA, USA). The consensus sequence was then re-called based on BWA-MEM mapping of trimmed but un-normalized read data to the genome scaffold and parsing of the mpileup alignment using in-house software. As a final step, LoFreq [42] was used to estimate variant frequencies in the obtained genomic data. A graphic representation of all major steps included in the sample preparation and analyses is provided in Additional file 2: Figure S1. The obtained sequences were phylogenetically analyzed with closely related sequences of isolates deposited in GenBank using MEGA6 [43], as previously described [25].

## Results

### Nucleic acids quantification and libraries fragment size

The nucleic acid concentrations obtained at different steps throughout the preparation of the libraries for sequencing are summarized in Additional file 3: Table S2. The lowest detected RNA concentration was 2 ng/μl and the maximum was 55 ng/μl. After RNA purification, the RNA concentrations of five samples were below the detection limit of Qubit (250 pg/μl); however, these samples resulted in sufficient cDNA quantity to be further processed in library preparation. The generated libraries had a relatively narrow combined distribution of mean fragment lengths (mean 351 bp, standard deviation 30 bp, with 26 of 30 libraries within the range of 334 to 371 bp) (see Additional file 3: Table S2). It was observed that the true fragment length distributions observed post-sequencing were shorter than expected based on Bioanalyzer reports, even after counting for adapter length (Table S2). As a result, a large proportion (more than 90% in nearly all libraries) of read pairs overlapped at the ends. The source of the discrepancy with the Bioanalyzer estimates is still unclear.

### Summarized statistics of the sequencing run

A summary of the sequencing run statistics as estimated by the MiSeq instrument is provided in Table 1. A cluster density of 917 +/- 19 K/mm^2^ and 92.34% of the clusters passing the chastity filter yielded a total of 8.4 Gigabases of data. Of 17.7 million total reads, 96.31% passed the instrument quality control filter. Almost 80% of the bases were assigned Phred quality scores equal or greater to Q30 (Q30 score is equivalent to an expected error rate of 0.001). The fraction of reads in the pool assigned to each sample varied from 0.0007 to 7.16% (mean 3.2 ± 1.4%).

**Table 1:** Statistics of next-generation sequencing of 30 avian paramyxovirus isolates in a single run

Data	Results
Cluster density (K/mm^2^)^a^	917 +/- 19
Clusters passing filter^b^	92.34%
Total number of reads	17762176
Pass-filter reads^c^	16403251
Percentage of reads passing filter	96.31%
≥ Q30^d^	77.9%
Lowest representation for any index^e^	0.0007%
Highest representation for any index^e^	7.16%

^a^ shows number of clusters per square millimeter (optimal cluster density is 1000–1200, can vary with chemistry)

^b^ indicates the purity of the signals detected from the clusters (i.e. signals passing chastity filter that is the ratio of the brightest base intensity divided by the sum of the brightest and second brightest base intensities and the filtration process removes the least reliable clusters from the image analysis results)

^c^ reads passing filter (about 15 million reads are expected from an optimally clustered flow cell)

^d^ percentage of bases with Phred quality score equal or greater to 30

^e^ percentage of pass-filter reads assigned to any index

### Optimization of the assembly/analysis workflow

**Fig. 2:**
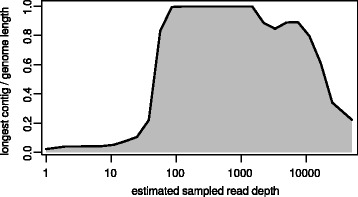
Analysis of Newcastle disease virus genome assembly at various read depths. Shown are the longest contig produced at each read depth as a fraction of the full genome length. Subsamples up to 200x were generated using digital normalization. Above 200x, additional reads were added using random subsampling (due to issues with high median cutoffs in the kh-mer package). At each subsampling depth, the final velvetg assembly was optimized for maximum contig length based on the “cov_cutoff” parameter

In order to take advantage of the overlapping reads, a merging step was introduced to produce longer pseudo-reads and to reduce complexity of the assembly task. An essential optimization was made by reducing the estimated coverage depth to a level that would still produce optimal assemblies. Two techniques for data reduction were investigated. Random sub-sampling resulted in loss of specific regions in the genome with reproducibly low coverage (data not shown). Digital normalization, which aims to down-sample high-coverage regions while preserving reads from low-coverage areas, provided means for decreasing the number of used reads to an optimal level without loss of data, and thus, was incorporated into the customized Galaxy workflow prior to assembly. In order to determine an optimal target depth for assembly, preliminary test assemblies using the Velvet assembler v1.2.10 [44] were performed on a geometric progression of sampling depths from 10x to 10000x (the approximate depth of the raw data) with an additional optimization of the velvetg “cov_cutoff” parameter for each depth (parameter used to low coverage nodes). The results indicated that optimal (in this case, full-length) assembly occurred over a range of approximately one order of magnitude (100x to 1000x). Below and above this range, fragmentation began to occur (Fig. 2).

### Data analysis

The final outputs of the analysis workflow for each sample included a consensus genome scaffold (.fasta), a file of all assembled contigs (.fasta), a variant frequency call file (variant call format or .vcf) and a set of summary statistics on the run and the assembly. An in-depth summary of the outputs from all samples is presented in Tables 2 and 3, including detailed information on read quality and depth distributions and genome coverage per sample. A total of 29 full-length or near-full-length APMV genomes (99.56% mean genome coverage) were obtained from 30 libraries with only one sample (#1005) having coverage below 99% and nine samples having 100% coverage (Tables 2 and 3). The lower and upper quartiles of median depth per position of the sequencing results were 2984 and 6894 respectively, allowing for accurate detection of low-frequency single nucleotide variants. In fact, all but one NDV samples had a median read depth of at least 2583 (the exception, sample 1005, was found to consist of approximately 98% avian influenza virus reads after host filtering). In addition to NDV genome assembly, the *de novo* strategy allowed for the detection of full-length and near-full-length genomes of avian influenza virus (AIV) in libraries of isolates 998, 1005, 1009 and 1011 [45], as well as infectious bronchitis virus (IBV) in samples 1003 and 1009. The coverage of the two obtained IBV genomes was 85.78 and 99.37%, while the sequenced AIV genes had coverages ranging between 92.23 and 100%, and two complete AIV genomes were sequenced (see Table 3). The estimated median depths for the IBV (5 and 22) and AIV (from 35 to 1274) isolates were lower (Table 3), most likely reflecting the lower titer of these viruses in the samples. Sample 959 was identified as a member of the novel APMV serotype 13 and the median depth for this sample was 3484. The host reads were between 0.1 and 5.4% (average 1.3%) of all reads per sample. No data was obtained from the library of sample 688 (only 0.0007% of the raw reads were assigned to this sample). The results from the comparison of the three NDV libraries prepared side-by-side with and without the capture step showed identical accuracy, not significantly affected overall coverage and near full-length and full-length genomes were obtained using both approaches. However, the number of NDV-specific reads decreased by approximately 30% when the capture step was not performed (see Table 4).

**Table 2:** Summary of sequencing and assembly data of 25 avian paramyxovirus isolates

Isolate number	% PF reads^a^	Number of raw read pairs	Number of filtered read pairs^b^	Forward read quality^c^	Reverse read quality^c^	Identified virus	Final coverage depth^c^	Number of reads used for consensus^d^	Consensus nucleotide length	Missing positions at 5' end^e^	Length of internal gaps	Missing positions at 3' end^e^	Percent coverage^e^
1002	2.49	409193	405137	2|37|38|38|38	2|36|37|38|38	NDV	0|3680|6088|7868|18004	390740	15124		68		99.55
1004	2.67	437755	432361	2|37|38|38|38	2|34|37|38|38	NDV	0|4185|6151|7909|14329	422150	15125		67		99.56
1007	4.20	688524	681691	2|37|38|38|38	2|36|37|38|38	NDV	0|817|3648|7368|19348	665220	15125		67		99.56
994	1.39	227500	226196	2|37|38|38|38	2|36|37|38|38	NDV	0|1758|2756|4186|14276	219609	15121		71		99.53
995	1.38	226050	224416	2|36|37|38|38	2|34|36|37|38	NDV	0|2162|2995|4197|9101	216240	15110		82		99.46
996	1.53	251238	250338	2|37|38|38|38	2|34|37|37|38	NDV	0|2383|3175|4411|9167	242158	15104	20	68		99.42
1001	3.79	621655	618002	2|37|38|38|38	2|36|37|38|38	NDV	0|4653|6784|9510|21623	594361	15122		70		99.54
997	1.72	281376	266251	2|37|38|38|38	2|35|37|38|38	NDV	0|1937|3026|4030|9645	233775	15104	22	66		99.42
999	1.70	279207	272105	2|37|38|38|38	2|36|37|38|38	NDV	0|1728|2583|4496|11915	241105	15108		84		99.45
1000	1.74	285079	280596	2|37|38|38|38	2|35|37|38|38	NDV	0|2338|3107|4441|8796	241724	15117		75		99.51
959	3.60	590494	588212	2|37|38|38|38	2|37|38|38|38	APMV-13	1|2250|3484|5801|23998	531520	16126	20			99.88
960	4.34	711909	709377	2|37|37|38|38	2|37|37|38|38	NDV	0|3738|5631|7347|19491	628837	15135	25	32		99.62
961	4.61	756197	753487	2|36|37|38|38	2|35|37|38|38	NDV	0|4585|6509|8206|14939	674667	15167	25			99.84
962	3.35	548833	547622	2|37|38|38|38	2|35|37|38|38	NDV	2|4617|6340|8772|14719	529553	15192				100.00
967	3.71	607876	597902	2|37|38|38|38	2|36|37|38|38	NDV	2|2673|3928|6881|26072	562657	15192				100.00
968	4.46	731692	727838	2|37|38|38|38	2|35|37|38|38	NDV	0|2507|4136|7393|22877	636218	15167	19			99.87
695	7.16	1156516	1129415	2|36|37|38|38	2|35|37|38|38	NDV	2|1678|3827|6301|32862	955829	15192				100.00
714	3.98	653603	643246	2|37|37|38|38	2|36|37|38|38	NDV	1|2082|3513|6724|35052	570825	15192				100.00
715	3.63	594802	580223	2|37|38|38|38	2|36|37|38|38	NDV	10|2608|5633|8881|28809	526885	15192				100.00
720	4.05	663757	657982	2|37|37|38|38	2|35|37|38|38	NDV	13|3821|6267|9252|27439	525313	15192				100.00
861	3.51	576077	574864	2|37|38|38|38	2|35|37|38|38	NDV	6|3930|6419|8881|27153	559394	15192				100.00
867	4.11	673510	668781	2|37|38|38|38	2|36|37|38|38	NDV	0|4284|6079|9028|23441	647586	15176	16			99.89
892	3.92	642753	642250	2|37|38|38|38	2|35|37|38|38	NDV	1|5147|7049|9922|18221	618591	15180	12			99.92
913	4.06	665640	661350	2|36|37|38|38	2|35|37|38|38	NDV	0|2278|3784|7011|31842	566280	15192				100.00
688	0.01	no data	NA	NA	NA	NA	NA		NA	NA	NA	NA	NA

*NA* not applicable

^a^ the fraction of reads assigned to each sample out of all number of reads that passed filter (i.e. pass-filter reads)

^b^ the number of paired reads remaining after host and internal control filtering

^c^ numbers represent distribution (minimum | lower quartile | median | upper quartile | maximum)

^d^ numbers of paired reads used to re-call the final consensus for each sequence

^e^ the missing nucleotides at the ends and the fraction of the expected full genome length covered by the consensus scaffold (i.e. not containing unknown nucleotides)

**Table 3:** Summary of sequencing and assembly data of five samples that were identified to have mixed populations of Newcastle disease virus (NDV) and other avian viruses

Isolate number	% PF reads^a^	Number of raw read pairs	Number of filtered read pairs^b^	Forward read quality^c^	Reverse read quality^c^	Identified virus	Final coverage depth^c^	Number of reads used for consensus^d^	Consensus nucleotide length	Missing positions at 5' end^e^	Length of internal gaps	Missing positions at 3' end^e^	Percent coverage^f^
*1003*	*3.33*	*546797*	*540519*	*2|37|38|38|38*	*2|36|37|38|38*	*NDV*	*0|4229|6090|8762|18055*	*525597*	*15127*		*65*		*99.57*
						*IBV* ^*g*^	*0|2|5|8|44*	*904*	*23711*	*269*	*3644*	*18*	*85.78*
*1005*	*3.44*	*564101*	*123024*	*2|36|37|38|38*	*2|33|36|37|38*	*NDV* ^*h*^	*0|7|13|19|36*	*1161*	*14494*	*343*	*272*	*83*	*95.41*
						*AIV* ^*i*^ *- PB2*	*2|685|1003|1446|2774*	*16804*	*2283*				*100.00*
						*AIV - PB1*	*2|1845|2896|3432|4634*	*38261*	*2334*				*100.00*
						*AIV - PA*	*68|1180|1496|1970|4721*	*23466*	*2151*				*100.00*
						*AIV - HA*	*12|595|1057|1598|2604*	*14687*	*1683*				*100.00*
						*AIV - NP*	*25|886|1306|1867|2563*	*11252*	*1497*				*100.00*
						*AIV - NA*	*11|416|838|1030|1692*	*7707*	*1410*				*100.00*
						*AIV - M1, M2*	*11|210|811|1357|2195*	*5699*	*982*				*100.00*
						*AIV - NEP, NS1*	*15|396|787|1222|1707*	*3796*	*838*				*100.00*
*1009*	*3.21*	*526425*	*519854*	*2|37|38|38|38*	*2|36|37|38|38*	*NDV*	*0|4216|6887|9403|18079*	*485669*	*15127*		*65*		*99.57*
						*IBV*	*0|15|22|35|92*	*3743*	*27469*	*136*	*19*	*18*	*99.37*
						*AIV - PB2*	*0|137|175|272|545*	*2791*	*2283*				*100.00*
						*AIV - PB1*	*3|370|651|835|1177*	*7692*	*2324*	*20*			*98.99*
						*AIV - PA*	*0|88|117|183|446*	*1675*	*2151*				*100.00*
						*AIV - HA*	*0|113|256|342|578*	*2399*	*1683*				*100.00*
						*AIV - NP*	*0|145|277|333|523*	*2117*	*1485*				*100.00*
						*AIV - NA*	*4|118|225|317|637*	*2226*	*1410*				*100.00*
						*AIV - M1,M2*	*2|46|132|183|246*	*793*	*958*	*24*			*96.71*
						*AIV - NEP, NS1*	*0|47|83|103|139*	*368*	*838*				*100.00*
*1011*	*3.44*	*565083*	*538171*	*2|37|38|38|38*	*2|36|37|38|38*	*NDV*	*1|3272|5386|7121|16101*	*499378*	*15192*				*100.00*
						*AIV - PB2*	*0|181|290|462|1254*	*6242*	*2283*				*100.00*
						*AIV - PB1*	*2|323|482|609|1077*	*7728*	*2334*				*100.00*
						*AIV - PA*	*8|127|172|220|437*	*2743*	*2151*				*100.00*
						*AIV - HA*	*3|217|449|685|906*	*5306*	*1683*				*100.00*
						*AIV - NP*	*3|180|380|605|758*	*4314*	*1497*				*100.00*
						*AIV - NA*	*3|74|98|153|242*	*1383*	*1410*				*100.00*
						*AIV - M1,M2*	*4|59|255|372|579*	*2092*	*982*				*100.00*
						*AIV - NEP, NS1*	*3|29|70|137|216*	*516*	*808*				*100.00*
*998*	*1.80*	*295662*	*294217*	*2|37|38|38|38*	*2|35|37|38|38*	*NDV*	*0|2248|3275|4719|11528*	*280492*	*15103*	*20*	*69*		*99.41*
						*AIV - PB2*	*0|22|33|48|78*	*477*	*2246*			*34*	*98.51*
						*AIV - PB1*	*0|34|56|85|125*	*698*	*2217*			*60*	*97.36*
						*AIV - PA*	*2|28|55|106|178*	*767*	*2141*	*10*			*99.53*
						*AIV - HA*	*0|9|18|29|53*	*204*	*1643*			*40*	*97.65*
						*AIV - NP*	*0|24|43|82|126*	*430*	*1456*	*35*		*6*	*97.26*
						*AIV - NA*	*1|21|28|41|83*	*272*	*1304*	*31*		*75*	*92.23*
						*AIV - M1,M2*	*0|8|28|65|87*	*196*	*923*	*56*			*92.62*
						*AIV - NEP, NS1*	*0|9|17|28|40*	*94*	*810*				*100.00*

^a^ the fraction of reads assigned to each sample out of all number of reads that passed filter (i.e. pass-filter reads)

^b^ the number of paired reads remaining after host and internal control filtering

^c^ numbers represent distribution (minimum | lower quartile | median | upper quartile | maximum)

^d^ numbers of paired reads used to re-call the final consensus for each sequence

^e^ for avian influenza viruses, the missing nucleotides refer to the beginning and the end of the coding sequences of the genes

^f^ the fraction of the expected full genome length covered by the consensus scaffold (i.e. not containing unknown nucleotides), for avian influenza genes, the coverage represents comparison to the coding sequences of the genes only

^g^ Infectious bronchitis virus

^h^ coverage depth and number of reads used to re-call the final consensus for this NDV isolate were impacted by the presence of influenza virus A in the sample (influenza reads were estimated to be approximately 98% of all reads, data not shown)

^i^ Avian influenza virus; PB2 = segment 1 polymerase PB2; PB1 = segment 2 polymerase PB1; PA = segment 3 polymerase PA; HA = segment 4 hemagglutinin; NP = segment 5 nucleocapsid protein; NA = segment 6 neuraminidase; M1, M2 = segment 7 matrix protein 1 and matrix protein 2; NEP = segment 8 nuclear export protein and nonstructural protein 1

**Table 4:** Comparison of differences in number of reads and genome coverage of three samples prepared with and without capture of NDV RNA

Virus designation	Number of reads		% fewer reads without capture	Identity of consensus sequences	Missing sequences at genome termini and internal gaps (in number of nucleotides)					
	With capture	Without capture			With capture			Without capture		
					5′	gaps	3′	5′	gaps	3′
691	403515	283501	29.7	100%	20	0	0	26	0	0
698	363962	262452	27.9	100%	0	0	0	25	0	0
901	415661	285405	31.3	100%	0	94	0	22	84	0

While the high-throughput workflow sometimes resulted in short segments of missing data at the genome termini and/or at one short internal gap, complete sequences for all coding regions of the 29 APMV positive samples were obtained directly from the workflow. Nearly all of the short missing regions occurred at either the termini (a common issue in viral NGS sequencing) [46] or at one specific intergenic location in the genome between genes N and P which displayed extremely low coverage in all analyzed samples (possibly as a result of high GC content – 76%). For the purpose of submitting full-length NDV sequences to GenBank, we sequenced the termini using a previously described protocol [47] and primers designed for the current study (see Additional file 4: Table S3). The internal gaps, where necessary, were sequenced using PCR and Sanger sequencing (for primers sequences see Additional file 4: Table S3). This additional work was not included in the time/cost estimates, as it was performed to submit complete NDV sequences to GenBank and would not be necessary for a full analysis of the coding regions.

### Time and cost estimates

The time and cost estimates for all steps are summarized in Additional file 5: Table S4. Assuming the addition of the first reagent as the start and the final dilutions of the samples as the end of the procedure, the approximate time taken for preparing 30 samples was 25 to 30 person-hours. The sequencing run (500 cycle kit) lasted 39 h. Submission of the raw data to the customized Galaxy workflow and data analysis on the cluster took an additional 2 to 3 h. The average cost of all steps, including all reagents but excluding labor, depreciation and maintenance of equipment, was estimated to be approximately 106 USD per sample.

## Discussion

Next-generation sequencing has been previously described for whole-genome sequencing of NDV by our team and others [48–55]; however, this study is the first report that demonstrates robust simultaneous genomic characterization of multiple NDV viruses in a single NGS run. The study further demonstrates the added benefit of conducting random non-targeted sequencing with an optimized *de novo* assembly workflow for identification of mixed viral infections. In contrast to previous work, here an optimized and customized workflow that employs publically available tools and produced consistently high quality assemblies of complete genomes is described in details. This study also provides detailed statistical and sequencing information that allows quality and quantity assessment of the obtained results.

Our findings demonstrate that the described chemistry and bioinformatics approach is sufficiently robust to obtain and distinguish the complete genomes of completely different types of RNA viruses during a mixed infection. In addition to the conclusive results with NDV and APMV-13 (family *Paramyxoviridae*), the complete or near complete genomes of four avian influenza and two infectious bronchitis viruses, which were co-infecting five samples originally identified as Newcastle disease viruses alone, were also obtained. Infectious bronchitis viruses belong to the family *Coronaviridae* and are single-stranded positive-sense RNA viruses with genome size of approximately 27,5 to 28 kb, excluding the poly (A) tail, which includes ten open reading frames [56]. The avian influenza viruses belong to the family *Orthomyxoviridae* and have genomes containing eight segments of single-stranded, negative-sense RNA that code for 10 or 11 proteins, depending on the strain [57]. Despite the diverse nature of the RNA present in samples with mixed populations, the procedure described here successfully produced complete genomes of these viruses.

Our results also demonstrate the capability of the methodology to produce quality libraries and assemblies without any physical or mechanical enrichment. The cDNA and dsDNA concentrations were not found to be proportional to the initial total RNA concentrations. The introduced nuclease step aided digestion of host nucleic acids resulting in low average number (see Tables 2 and 3) of host-associated reads per sample. The abundance of host nucleic acids may pose a problem in obtaining sufficient numbers of viral reads for optimal viral genome assembly [4]. To avoid or decrease problems caused by contamination with host sequences others have developed methodologies for enrichment of target viral RNAs. We have not utilized any pretreatment or purification; however, a target-specific capture step with biotinylated oligos designed from three different conservative regions of the NDV genome was tested. The comparison of results from samples with and without the RNA capture step presented here demonstrates that the primary tradeoff comes in the form of approximately 30% reduced depth of coverage, although the coverage was still sufficient for proper consensus re-calling (see Table 4). The ability of the capture step to reduce host sequences and other non-target RNA and to improve downstream assembly and analysis should be further assessed on different sample types (e.g. clinical samples, formalin-fixed paraffin-embedded samples) that may contain less amounts of viral RNA. For egg-grown viruses with high viral titers the observed decrease of reads without the RNA capture step was not essential for obtaining complete coverage with sufficient depth. In clinical diagnostic samples, however, the number of NDV sequencing reads is often significantly lower, and introducing the RNA capture step could improve the final results.

There is a clear difference between the presented application of NGS and the use of this approach in diagnostics. Here we describe the use of high-titer egg-grown viruses for production of high quality and deep data useful for detailed genomic characterization and rare variant analyses. However, the use of this NGS technology for diagnostics is more complex. It requires to clearly establish sensitivity, specificity and limit of detection based on the nature of samples and these are beyond the scope of the current work. The described methodology has been successfully transferred for use with clinical samples and optimization studies are in progress in our lab.

Prior to the production run, considerable time was spent optimizing the assembly and analysis workflow for the task at hand. While some of the steps in the workflow are fairly standard procedures in NGS analyses (QC summarization, adapter trimming, contaminant read filtering), others were tailored to the specific characteristics of the data being generated. The most critical optimization, however, was reducing estimated coverage depth to a level that would produce optimal assemblies. It has previously been shown that, past a certain level, increasing read depth can decrease *de novo* assembly quality [58]. This effect can have significant consequences when working with massively deep sequencing data such as viral population studies that can easily exceed 10000x sample coverage. Digital normalization has been included in similar workflows by others [59, 60] but is often overlooked in naïve approaches to high-coverage *de novo* assembly. As the assembler used in our workflow (MIRA) is relatively resource-intensive overlap-layout-consensus (OLC) assembler, we chose a target (100x) at the lower end of the empirically determined optimal range to incorporate as a cut-off into the customized production workflow. Graph-based assemblers such as Velvet utilize de Bruijn graph algorithms and assemble data by representing the genome by a set of short k-mer sequences [44]. Notably, graph-based assemblers are less resource-demanding and can be successfully utilized with limited computational resources. However, for graph-based assemblers, the k-mer size is an essential parameter [44, 59, 60] and the optimal value has to be determined depending on the characteristics of the sequence reads, while this is avoided using an OLC assembler. In our hands, MIRA consistently produces quality assemblies with minimal tuning needed. Additionally, due to the potential skewing effects of digital normalization and V-FAT scaffolding on the proportion of nucleotide variant frequencies, the post-assembly step to re-map the un-normalized data to the genome scaffold allowed for proper consensus re-calling and precise variant analysis.

One important aspect to the use of NGS approaches in mainstream viral sequencing studies is the capacity to multiplex samples in order to reduce costs. The time and cost summary for all steps (summarized in Additional file 5: Table S4) demonstrate that the simultaneous processing of 30 samples requires approximate one hour of operator time per sample with a cost of approximately 106 USD per sample. Those values, although still high for diagnostics purposes, are 15 to 20 times lower (based on internal estimates) compared to the cost of primer-based sequencing with Sanger technologies. Furthermore, the demonstrated lower and upper quartiles of median depth per position (2984 and 6894, respectively), allow for accurate consensus re-calling and rare variant analysis. The final output not only includes the consensus genome sequence but also produced a variant call format file (https://samtools.github.io/hts-specs/VCFv4.2.pdf) and demonstrate that the protocol could be used for research on viral quasispecies and evolutionary studies (Dimitrov et al., in preparation). As previously reported by Gould et al., the 1998 Newcastle disease outbreak in Australia was preceded by several months of circulation of mutant quasispecies of the virulent cleavage site [61]. These viral variants were undetected in the Sanger consensus sequences but could have been detected and properly quantified if the methodology described here was available at that time. The obtained genomic coverages (see Tables 2 and 3) illustrate the ability of the described protocol in generating full-length or near-full-length RNA virus genomes. Although very short internal gaps were present due to complete absence of coverage in the raw data and short sequences at the genome termini were missing, all coding sequences (commonly used in genetic studies) were obtained.

The total turnaround time for the entire testing (sample preparation, sequencing and analyses) was approximately 72 h, of which most of the time consisted of library preparation and the sequence run. The duration of the sequencing run could be reduced by approximately 15 h by using a 300-cycle configuration without any anticipated drop-off in assembly quality based on our observed fragment length distributions, although this assumption is untested. The time taken for data analysis may be expected to vary somewhat based on the available computational resources in a lab, although in our protocol this represents a small fraction of the total turnaround time to begin with.

The obtained results were phylogenetically consistent with preliminary studies of the tested viruses (data not shown) and expectations based on previous research. Almost all of the samples from Pakistan were of sub-genotype VIIi which is currently circulating in Pakistan and may be causing a new panzootic [25, 62]. Ukrainian samples were of different sub-genotypes (II, VIg and VIId) that have been reported to be isolated from pigeons in Ukraine [25] and also in Europe [63]. The Nigerian samples were of genotypes XIVb and XVIIa which have been reported to circulate in Nigeria since 2006 [64] and previously un-sequenced full-length genomes of these sub-genotypes have been reported by us [48, 49]. In addition, and demonstrating the broad applicability and the advantages of the *de novo* approach described here, the first complete APMV-13 genome was obtained [17] and avian influenza and infectious bronchitis viruses populations were identified. Phylogenetic analyses of the obtained NDV and IBV sequences are presented in Additional file 6: Figures S2 and S3).

## Conclusion

In summary, a robust chemistry and bioinformatics protocol utilizing publicly available tools to sequence and analyze complete genomes from small RNA viruses is described. Thirty-five full-length or near-full-length avian RNA viral genomes with a high median coverage depth were successfully sequenced out of 30 samples. The applied *de novo* approach allowed identification of mixed viral populations in some of the samples. The combination of multiplexing NGS technology with the customized Galaxy workflow platform enabled a quick turnaround time and cost-efficient methodology for simultaneous characterization of multiple viral genomes.

## Additional files

Additional file 1: Table S1. Background information of the avian paramyxovirus isolates used in this study. (DOCX 15 kb)

Additional file 2: Figure S1. Major processing steps used in the current study (PDF 222 kb)

Additional file 3: Table S2. Nucleic acid concentrations and library fragment size distributions of thirty virus isolates used in the study. (DOCX 18 kb)

Additional file 4: Table S3. Sequences of primers used for sequencing internal gaps and missing termini. (DOCX 13 kb)

Additional file 5: Table S4. Time and cost analysis of next-generation sequencing of thirty avian paramyxovirus isolates. (DOCX 15 kb)

Additional file 6: Figure S2. Phylogenetic analysis based on the complete genome coding sequence of Newcastle disease virus isolates studied here and selected closely related sequences from GenBank. **Figure S3.** Phylogenetic analysis based on the hypervariable region of the spike protein gene of Infectious bronchitis virus studied here and selected closely related sequences from GenBank. (PDF 142 kb)
