# Supplementary material for: A robust and cost-effective approach to sequence and analyze complete genomes of small RNA viruses
Source: Virol J. 2017 Apr 7;14:72. doi: 10.1186/s12985-017-0741-5 (PMC5384157; doi:10.1186/s12985-017-0741-5)
Supplement: Supplementary file 1 — Background information of the avian paramyxovirus isolates used in this study. (DOCX 15 kb) [file 12985_2017_741_MOESM1_ESM.docx]

**Table S1** Background information of the avian paramyxovirus isolates used in this study.

| **Isolate number** | **Host species** | **Host common name/type** | **Country** | **Isolate Name** | **Year of isolation** | **Genotype/**  **Serotype^a^** | **GenBank accession number** |
| --- | --- | --- | --- | --- | --- | --- | --- |
| 1002 | *Gallus gallus* | chicken | Pakistan | 1A | 2015 | VII i | KY076035 |
| 1003 | *Gallus gallus* | chicken | Pakistan | 2A | 2015 | VII i | KY076036 |
| 1004 | *Gallus gallus* | chicken/broiler | Pakistan | 5A | 2015 | VII i | KY076037 |
| 1007 | *Gallus gallus* | chicken/broiler | Pakistan | 6A | 2015 | VII i | KY076038 |
| 1005 | *Gallus gallus* | chicken/broiler | Pakistan | 10A | 2015 | VII i | KY076043 |
| 994 | *Gallus gallus* | chicken/broiler | Pakistan | 12A | 2015 | VII i | KY076030 |
| 1009 | *Gallus gallus* | chicken/layer | Pakistan | 13A | 2015 | VII i | KY076039 |
| 995 | *Gallus gallus* | chicken/layer | Pakistan | 15A | 2015 | VII i | KY076031 |
| 996 | *Columba livia* | wild pigeon | Pakistan | 20A | 2015 | VII i | KX496962 |
| 1001 | *Columba livia* | pigeon | Pakistan | 22A | 2015 | VII i | KX496963 |
| 997 | *Columba livia* | pigeon | Pakistan | 23A | 2015 | VII i | KX496964 |
| 1011 | *Columba livia* | Pigeon | Pakistan | 25A | 2015 | VI k | KX236101 |
| 998 | *Gallus gallus* | chicken/broiler | Pakistan | 26A | 2011 | VII i | KY076032 |
| 999 | *Gallus gallus* | chicken/layer | Pakistan | 27A | 2015 | VII i | KY076033 |
| 1000 | *Gallus gallus* | chicken/broiler | Pakistan | 30A | 2015 | VII i | KY076034 |
| 959 | *Anser albifrons* | White-fronted Goose | Ukraine | Askania-Nova/48-15-02 | 2011 | APMV-13 | KX119151 |
| 960 | *Gallus gallus* | chicken | Ukraine | Kharkiv/66 | 2007 | VII d | KU295453 |
| 961 | *Gallus gallus* | chicken | Ukraine | Lyubotyn | 2003 | VII d | KU295454 |
| 962 | *Gallus gallus* | chicken | Ukraine | Bashtanivske/20-02 | 2013 | VII d | KU295455 |
| 967 | *Columba livia* | pigeon | Ukraine | Kharkiv/23-01 | 2013 | VI g | KY042127 |
| 968 | *Columba livia* | pigeon | Ukraine | Doneck/3 | 2007 | II | KU133362 |
| 695 | *Anas platyrhynchos* | duck/domestic | Nigeria | KG/LOM/11-16 (N11) | 2009 | XIV b | KT948996 |
| 714 | *Gallus gallus* | chicken | Nigeria | VRD09/001 (N19) | 2009 | XIV b | KY171994 |
| 715 | *Gallus gallus* | chicken | Nigeria | VRD09/031 (N23) | 2009 | XIV b | KY171993 |
| 720 | *Gallus gallus* | chicken | Nigeria | KD/TW/03T (N45) | 2009 | XIV b | KY171990 |
| 861 | *Coturnix japonica* | quail | Nigeria | VRD17/04 (N2) | 2004 | XVII a | KY171991 |
| 867 | *Gallus gallus* | chicken | Nigeria | VRD124/06 (N11) | 2006 | XVII a | KY171995 |
| 892 | *Gallus gallus* | chicken | Nigeria | JN/469 (N44) | 2009 | XVII a | KY171992 |
| 913 | *Gallus gallus* | chicken | Nigeria | VRD10/143 (N68) | 2010 | XIV b | KY171989 |
| 688 | *Columba livia* | pigeon | Nigeria | ZM/KN/PG01 (N1) | 2009 | XVII a | KC568204 |

^a^ all isolates except 959 were identified as members of APMV-1 prior to next-generations sequencing
