# Supplementary figures and images for: A robust and cost-effective approach to sequence and analyze complete genomes of small RNA viruses
Source: Virol J. 2017 Apr 7;14:72. doi: 10.1186/s12985-017-0741-5 (PMC5384157; doi:10.1186/s12985-017-0741-5)

**Additional file 2, Fig. S1** Major processing steps used in the current study

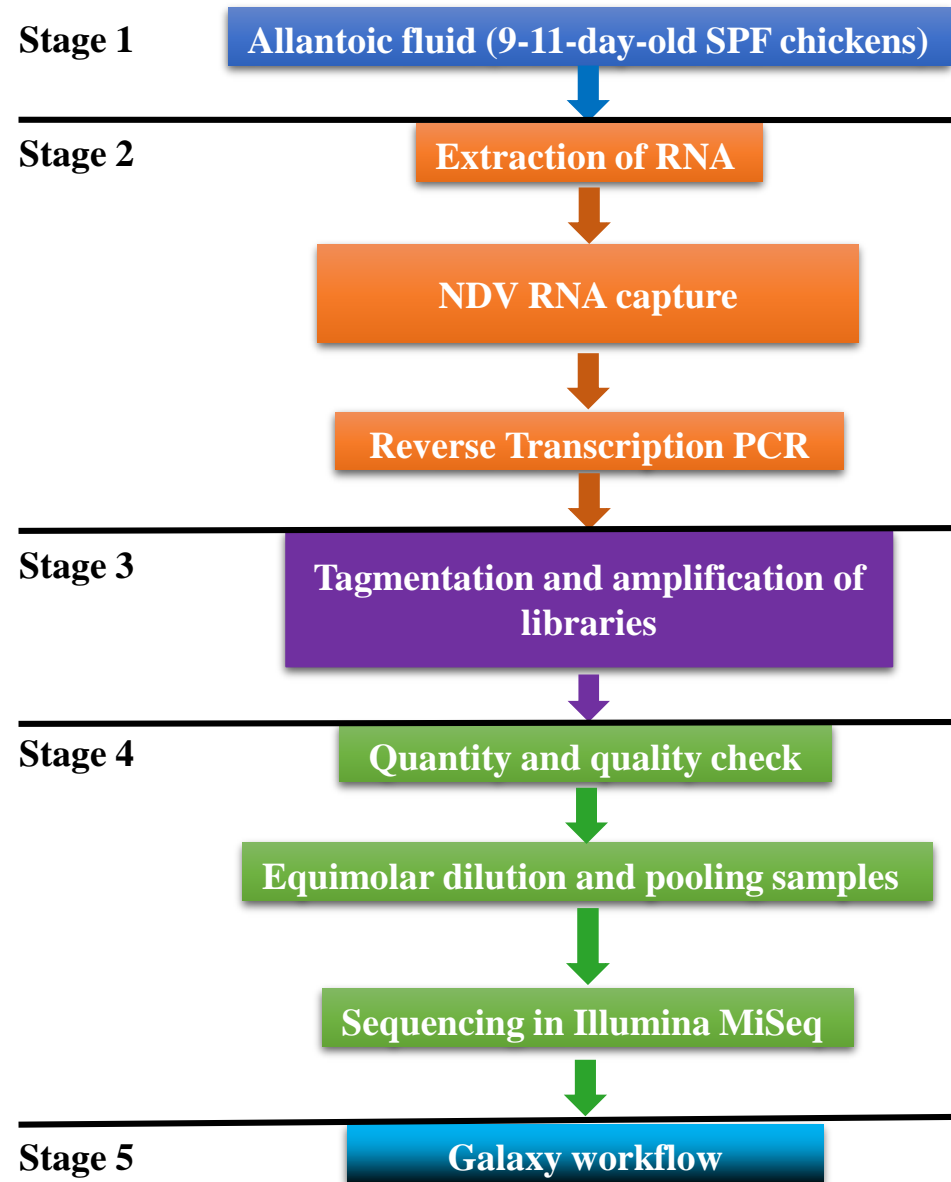

Supplement: Supplementary file 2 — Major processing steps used in the current study (PDF 222 kb) [file 12985_2017_741_MOESM2_ESM.pdf]
