# Supplementary material for: A robust and cost-effective approach to sequence and analyze complete genomes of small RNA viruses
Source: Virol J. 2017 Apr 7;14:72. doi: 10.1186/s12985-017-0741-5 (PMC5384157; doi:10.1186/s12985-017-0741-5)
Supplement: Supplementary file 3 — Nucleic acid concentrations and library fragment size distributions of thirty virus isolates used in the study. (DOCX 18 kb) [file 12985_2017_741_MOESM3_ESM.docx]

**Table S2** Nucleic acid concentrations and library fragment size distributions of thirty virus isolates used in the study.

| **Isolate number** | **Concentrations in ng/µl** | | | **Mean fragment length (standard deviation)** | |
| --- | --- | --- | --- | --- | --- |
|  | **RNA** | **cDNA** | **dsDNA** | **Bioanalyzer^a^** | **Fast QC^b^** |
| 1002 | 9.68 | 11.70 | 1.14 | 335 | 228 (104) |
| 1003 | 21.40 | 10.10 | 2.74 | 340 | 186 (90) |
| 1004 | 5.08 | 3.81 | 2.65 | 350 | 210 (90) |
| 1007 | 3.81 | 6.57 | 5.04 | 365 | 182 (79) |
| 1005 | 25.90 | 0.25 | 1.10 | 335 | 173 (81) |
| 994 | 55.00 | 10.70 | 1.17 | 340 | 216 (98) |
| 1009 | 3.95 | 1.20 | 1.99 | 350 | 207 (89) |
| 995 | 10.60 | 7.39 | 1.81 | 350 | 223 (104) |
| 996 | 15.30 | 15.20 | 1.26 | 360 | 214 (101) |
| 1001 | 6.88 | 6.71 | 2.19 | 340 | 177 (92) |
| 997 | 41.20 | 0.67 | 2.78 | 340 | 202 (102) |
| 1011 | 7.16 | 0.59 | 1.50 | 340 | 169 (79) |
| 998 | 11.20 | 1.49 | 2.72 | 340 | 200 (104) |
| 999 | 46.80 | 1.87 | 2.14 | 345 | 201 (98) |
| 1000 | 27.80 | 1.94 | 2.12 | 345 | 204 (92) |
| 959 | 2.20 | 0.34 | 2.61 | 303 | 124 (71) |
| 960 | 2.00 | 0.23 | 1.26 | 367 | 125 (79) |
| 961 | 6.19 | 4.19 | 1.54 | 371 | 131 (81) |
| 962 | 6.91 | 0.17 | 1.68 | 427 | 183 (97) |
| 967 | ND | 0.33 | 1.87 | 350 | 135 (72) |
| 968 | ND | 1.07 | 2.12 | 345 | 114 (67) |
| 695 | 33.80 | 1.12 | 4.10 | 360 | 125 (70) |
| 715 | 28.00 | 0.57 | 2.81 | 350 | 173 (78) |
| 714 | ND | 1.51 | 2.16 | 334 | 123 (73) |
| 720 | 33.5 | 0.84 | 2.42 | 458 | 203 (113) |
| 861 | 14.00 | 2.59 | 3.82 | 350 | 194 (89) |
| 867 | ND | 0.33 | 3.42 | 347 | 151 (69) |
| 892 | 10.80 | 5.34 | 4.97 | 365 | 184 (91) |
| 913 | ND | 1.18 | 2.05 | 345 | 123 (72) |
| 688 | 17.00 | 2.33 | 2.23 | 295 | NA |

ND = not detected (below the detection limit of 250 pg/µl)

NA = not applicable

^a^ as estimated by High Sensitivity DNA kit on the Agilent 2100 Bioanalyzer

^b^ as estimated from sequencing reads by Fast QC tool within Galaxy workflow
