# Supplementary material for: A robust and cost-effective approach to sequence and analyze complete genomes of small RNA viruses
Source: Virol J. 2017 Apr 7;14:72. doi: 10.1186/s12985-017-0741-5 (PMC5384157; doi:10.1186/s12985-017-0741-5)
Supplement: Supplementary file 4 — Sequences of primers used for sequencing internal gaps and missing termini. (DOCX 13 kb) [file 12985_2017_741_MOESM4_ESM.docx]

**Table S3** Sequences of primers used for sequencing internal gaps and missing termini.

| **Primer designation** | **Used to sequence samples** | **5ʹ – 3ʹ sequence** | **Nucleotide position** |
| --- | --- | --- | --- |
| 1302_F | 960, 994 to 1000, 1002 to 1005, 1007, 1009 | CYGCTGCCCAACGAGTATC | 1302 |
| 1951R | 960, 994 to 1000, 1002 to 1005, 1007, 1009 | ATGACAGTCCCACTGGTCTC | 1951 |
| 1316_F | 1001 | GTATCCGATGAGATTGGTACTG | 1316 |
| 1855_R | 1001 | ATCTTGGTAGCTCGTCCAGA | 1855 |
| 1R | 968 | AGAGATATGAGAGCACCYT | 330-348 |
| 7F | 1005 | CTTGGARGTCACAATACTRGG | 14713-14733 |
| 7R | 960, 961, 996, 997, 998, 1005 | CCGCAAARTTCCATCTATCCTC | 257-278 |
| 13R | 959 | TCCCATTGTTAAAGGCATCG | 334 |
| 19MER-1 | 968 | GATACTCGGATGCAGGTGC |  |
| 19MER-1-poly A(B) | 968 | GATACTCGGATGCAGGTGCAAAAAAAAAAAAAAAAAAAAAB |  |
| 19MER-1-poly T(V) | 968 | GATACTCGGATGCAGGTGCTTTTTTTTTTTTTTTTTTTTTV |  |
| 19MER-4-7 | 960, 961, 996, 997, 998, 1005 | CATTCTGCGATGCTCGATT |  |
| 19MER-4-7-poly A(B) | 960, 961, 996, 997, 998, 1005 | CATTCTGCGATGCTCGATTAAAAAAAAAAAAAAAAAAAAAB |  |
| 19MER-4-7-poly T(V) | 960, 961, 996, 997, 998, 1005 | CATTCTGCGATGCTCGATTTTTTTTTTTTTTTTTTTTTTTV |  |
| 19MER-13 | 959 | ACGTATCGGGCTGTTAAGG |  |
| 19MER-13-poly A(B) | 959 | ACGTATCGGGCTGTTAAGGAAAAAAAAAAAAAAAAAAAAAB |  |
| 19MER-13-poly T(V) | 959 | ACGTATCGGGCTGTTAAGGTTTTTTTTTTTTTTTTTTTTTV |  |
