# Supplementary material for: A robust and cost-effective approach to sequence and analyze complete genomes of small RNA viruses
Source: Virol J. 2017 Apr 7;14:72. doi: 10.1186/s12985-017-0741-5 (PMC5384157; doi:10.1186/s12985-017-0741-5)
Supplement: Supplementary file 5 — Time and cost analysis of next-generation sequencing of thirty avian paramyxovirus isolates. (DOCX 15 kb) [file 12985_2017_741_MOESM5_ESM.docx]

**Table S4** Time and cost analysis of next-generation sequencing of thirty avian paramyxovirus isolates.

| **Steps** | **Time (30 samples)** | **Cost in USD per sample** |
| --- | --- | --- |
| RNA extraction and concentration determination | 9-10 hours | 5 |
| NDV RNA capture and enrichment | 3-4 hours | 3 |
| Reverse transcription and cDNA concentration determination | 4-5 hours | 8 |
| Tagmentation and amplification | 3-4 hours | 35 |
| Determination of dsDNA concentration and fragment size | 3 hours | 4 |
| Equimolar dilution and pooling of libraries | 1 hours | - |
| Sequencing | 39 hours* | 51 |
| Post-run data processing and consensus assembly | 2-3 hours | - |
| Average | 25-30 hours (*excluding the sequencing run time) | 106 |
