# Supplementary material for: A robust and cost-effective approach to sequence and analyze complete genomes of small RNA viruses
Source: Virol J. 2017 Apr 7;14:72. doi: 10.1186/s12985-017-0741-5 (PMC5384157; doi:10.1186/s12985-017-0741-5)
Supplement: Supplementary file 6 — Phylogenetic analysis based on the complete genome coding sequence of Newcastle disease virus isolates studied here and selected closely related sequences from GenBank. Figure S3. Phylogenetic analysis based on the hypervariable region of the spike protein gene of Infectious bronchitis virus studied here and selected closely related sequences from GenBank. (PDF 142 kb) [file 12985_2017_741_MOESM6_ESM.pdf]

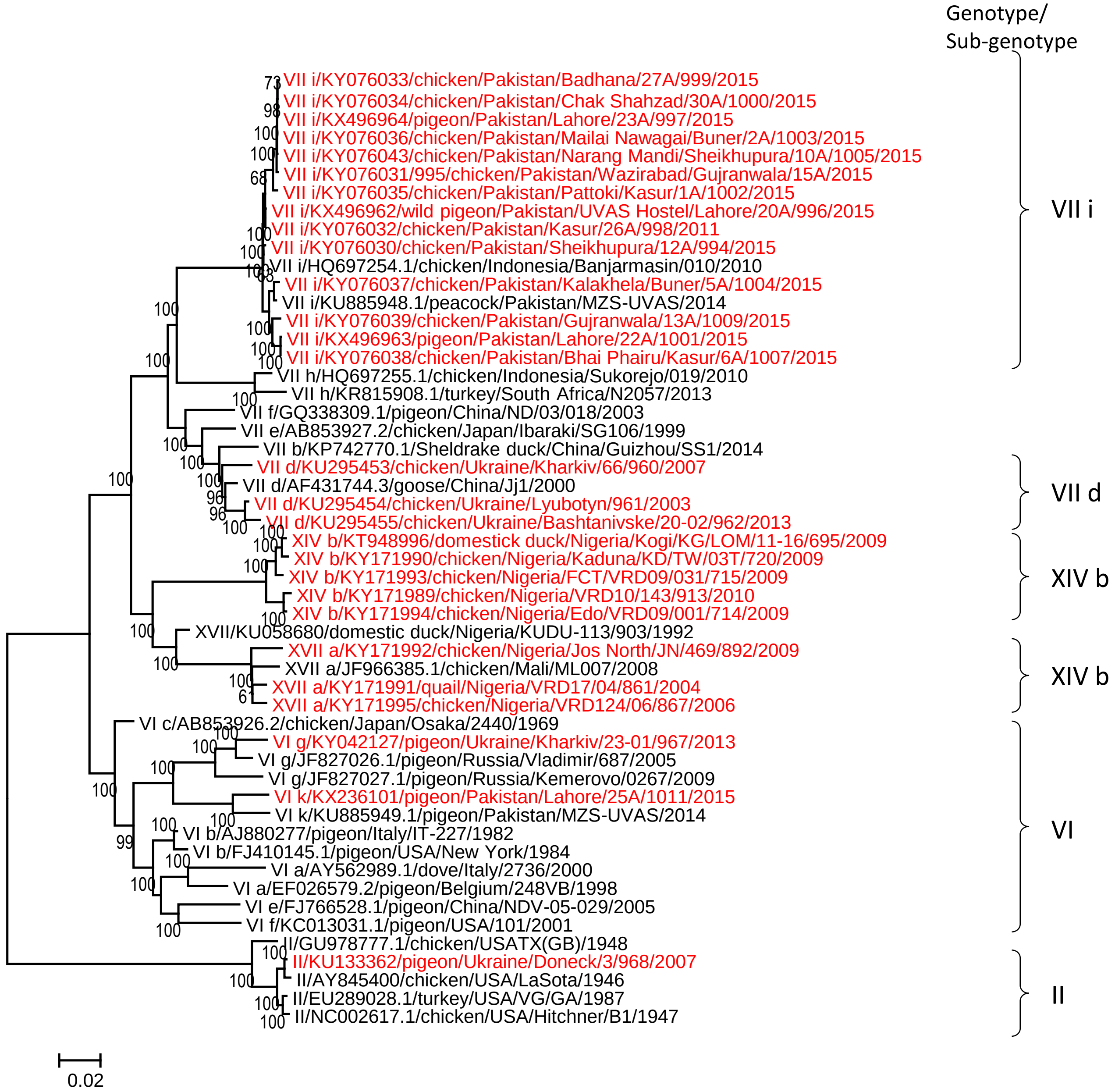

Fig. S2 Phylogenetic analysis based on the complete genome coding sequence of Newcastle disease virus isolates studied here and selected closely related sequences from GenBank.

All sequences (n = 52) were aligned using Multiple Alignment with Fast Fourier Transformation (MAFFT v7.017) [1] as implemented in the Geneious software v8.1.4 [2]. The coding regions of the complete genomes were used to construct the phylogenetic tree using MEGA6 [3]. To select best-fit substitution model, the Bayesian Information Criterion (BIC) and corrected Akaike Information Criterion (AICc) values were estimated using MEGA6. The General Time Reversible (GTR) model as implemented in MEGA6 with a discrete gamma distribution (4 categories (+*G*, parameter = 0.4992)) with 500 bootstrap replicates was used in the analysis. The rate variation model allowed for some sites to be evolutionarily invariable ([+*I*], 47.0931% sites). The tree with the highest log likelihood (-81841.1100) is shown. The branch lengths are proportional to the differences between the isolates. The codon positions included were the 1st, 2nd, 3rd, and noncoding and positions containing gaps and missing data were eliminated from the datasets. The analysis involved 52 nucleotide sequences. All positions containing gaps and missing data were eliminated. There were a total of 13507 positions in the final dataset. The sequences highlighted in red color were obtained in the current study.

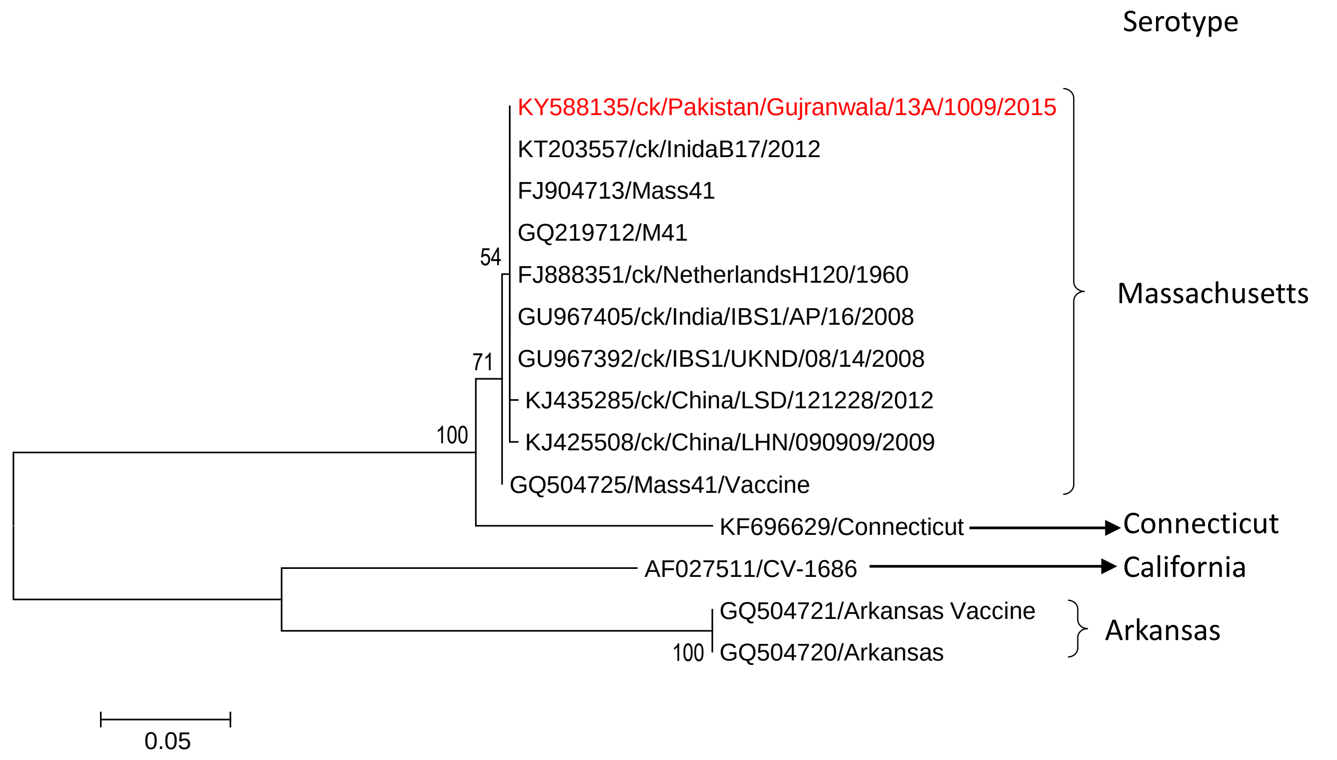

Fig. S3 Phylogenetic analysis based on the hypervariable region of the spike protein gene of Infectious bronchitis virus studied here and selected closely related sequences from GenBank.

All sequences (n = 14) were aligned using Multiple Alignment with Fast Fourier Transformation (MAFFT v7.017) [1] as implemented in the Geneious software v8.1.4 [2]. The hypervariable region of the spike protein genes were used to construct the phylogenetic tree using MEGA6 [3]. To select best-fit substitution model, the Bayesian Information Criterion (BIC) and corrected Akaike Information Criterion (AICc) values were estimated using MEGA6. The Tamura 3-parameter model as implemented in MEGA6 with a discrete gamma distribution (4 categories (+*G*, parameter = 0.08979)) with 500 bootstrap replicates was used in the analysis. The tree with the highest log likelihood (-1081.9953) is shown. The branch lengths are proportional to the differences between the isolates. The codon positions included were the 1st, 2nd, 3rd, and noncoding and positions containing gaps and missing data were eliminated from the datasets. The analysis involved 14 nucleotide sequences. All positions containing gaps and missing data were eliminated. There were a total of 330 positions in the final dataset. The sequence of KY588134/ck/Pakistan/Mailai Nawagai/Buner/2A/1003/2015 was not included in the analysis due to missing genetic data in the spike protein gene. The sequence highlighted in red color was obtained in the current study.

1. Katoh K, Misawa K, Kuma K, Miyata T. MAFFT: a novel method for rapid multiple sequence alignment based on fast Fourier transform. Nucleic Acids Res. 2002; 30:3059-66. http://dx.doi.org/10.1093/nar/gkf436
2. Kearse M, Moir R, Wilson A, Stones-Havas S, Cheung M, Sturrock S, Buxton S, Cooper A, Markowitz S, Duran C, et al. Geneious Basic: an integrated and extendable desktop software platform for the organization and analysis of sequence data. Bioinformatics. 2012; 28:1647-9. http://dx.doi.org/10.1093/bioinformatics/bts199
3. Tamura K, Stecher G, Peterson D, Filipski A, Kumar S. MEGA6: molecular evolutionary genetics analysis version 6.0. Mol Biol Evol. 2013; 30:2725-9. http://dx.doi.org/10.1093/molbev/mst197
